# Discrepancy in the Histological Diagnoses of Oral Lichen Planus Based on WHO Criteria Versus the Newly Proposed Diagnostic Set of the American Academy of Oral and Maxillofacial Pathology

**DOI:** 10.3390/diagnostics15050558

**Published:** 2025-02-26

**Authors:** Maria Zaharieva Mutafchieva, Desislava Atanasova Tashkova

**Affiliations:** 1 Department of Periodontology and Oral Mucosa Diseases, Faculty of Dental Medicine, Medical University of Plovdiv, 4000 Plovdiv, Bulgaria; 2 Department of General and Clinical Pathology, Faculty of Medicine, Medical University of Plovdiv, 4000 Plovdiv, Bulgaria; desislava.tashkova@mu-plovdiv.bg; 3 Department of Clinical Pathology, Oncology Centre—Plovdiv, Medical University of Plovdiv, 4000 Plovdiv, Bulgaria

**Keywords:** oral lichen planus, OLP, American Academy of Oral and Maxillofacial Pathology (AAOMP) diagnostic set, histological assessment

## Abstract

**Background/Objectives**: The diagnosis of oral lichen planus (OLP) is challenging because many other oral diseases demonstrate similar clinical and microscopic features. Clinicopathological discrepancy and inter- and intraobserver variability in the histological assessment of OLP have been shown in the literature, as there are no unified diagnostic criteria for the disease. In 2016, the American Academy of Oral and Maxillofacial Pathology (AAOMP) proposed a new diagnostic set for OLP. The aim of the study was to assess the reliability of the AAOMP histological criteria in diagnosing OLP. **Methods**: In this retrospective study, 34 histological sections, once diagnosed as OLP, were revised by a second pathologist using the WHO criteria. Then, all specimens were analyzed for the presence (P) or absence (A) of the criteria listed in the AAOMP diagnostic set. The reproducibility of the histological diagnosis of OLP when applying the different sets of diagnostic criteria was assessed. **Results**: From the AAOMP diagnostic criteria, hydropic degeneration was found in 35.2%, lymphocytic exocytosis in 32.3%, mild epithelial dysplasia in 2.9%, verrucous epithelial architectural change in 0% and band-like lymphocytic infiltrate, confined to the epithelium-lamina propria interface in 55.8% of the samples. Reproducibility of the histological diagnosis of OLP was achieved in only 19.3% of the cases when applying the 1978 WHO criteria versus the newly proposed AAOMP criteria. **Conclusions**: A large number of OLP cases failed to meet the AAOMP histological criteria in the present study. Further studies are needed to assess the validity of the proposed diagnostic set.

## 1. Introduction

A number of dermatological diseases can affect the oral mucosa. Lichen planus (LP) is a common mucocutaneous disease. Classic LP on the skin presents as polygonal pruritic red–purple papules, primarily affecting the extremities, wrists, ankles and lumbar region [1,2]. When oral lesions occur together with skin lesions, diagnosis is relatively straightforward, but in up to 25% of cases, the oral cavity is the only site of involvement—oral lichen planus (OLP) [1]. OLP has a global prevalence of 1.01% and usually affects women aged 30 to 60 years [3]. Although enormous efforts have been invested in research, there are still many unanswered questions regarding the etiology, pathogenesis and malignant potential of the disease. Clinically, OLP can manifest in six different forms—reticular, papular, plaque-like, atrophic, erosive or bullous [4]. Keratotic forms (reticular, papular, plaque-like) appear as white lesions that can not be rubbed off, while non-keratotic forms present either as red areas of thinned mucosa, erosions or blisters. Wickham striae, representing slightly raised gray–white lines in a net-like configuration, are considered a pathognomonic sign of the disease and should usually be present elsewhere in the oral cavity. Subjective symptoms range from mild discomfort to excruciating pain.

Cases of OLP often present difficulties in terms of differential diagnostic analysis. Due to the wide variety of clinical manifestations, OLP must be distinguished from a number of other white, red, red-white, ulcerative, and bullous lesions of the oral mucosa that may resemble OLP both clinically and histopathologically—the so-called oral lichenoid lesions (OLLs) [5]. On the gingiva, OLP often manifests as desquamative gingivitis—a situation in which it is clinically indistinguishable from some OLP mimics, such as mucous membrane pemphigoid (MMP), pemphigus vulgaris, etc. The following diagnostic criteria have been proposed as an attempt to increase the accuracy of the clinical diagnosis of OLP: detection of multiple lesions with bilateral symmetric distribution; erosive, atrophic, bullous and plaque-type lesions are only accepted as a subtype in the presence of reticular lesions elsewhere in the oral mucosa [6]; lesions are not localized exclusively in contact with dental restorations or in sites of smokeless tobacco placement; lesion onset does not correlate with the start of a medication or the use of cinnamon-containing products [5].

However, due to the high clinical mimicry with other conditions, histological analysis of suspected OLP lesions is generally advised. Moreover, OLP is defined as an oral potentially malignant disorder (OPMD) [7]. Histologically, OLP demonstrates typical but non-pathognomonic features. Therefore, the pathoanatomical diagnosis of the disease is not definitive but should be considered in correlation with the clinical diagnosis and accepted only when the two coincide. Making an accurate histological diagnosis is challenging due to the following factors: 1. The histological finding may vary depending on the clinical form of OLP, the biopsy area, the activity of the condition and the administration of immunosuppressive therapy; 2. No unified diagnostic criteria for the disease have been adopted; 3. There seems to be a low correlation in the diagnosis of oral lichen planus among examiners regardless of the set of histopathological diagnostic criteria used; 4. Many microscopic features of OLP are not specific to OLP and can be found in other diseases [5].

The histological definition of OLP was first formulated by the WHO in 1978. The proposed set of diagnostic criteria includes hyperortho- or hyperparakeratosis; acanthosis or epithelial atrophy; Civatte bodies in the basal layer of the epithelium or superficial lamina propria; liquefaction degeneration in the basal cell layer, eosinophilic material in the basement membrane, saw-tooth rete ridges and a well-defined band-like zone of inflammatory cells (mainly lymphocytes) confined to the superficial lamina propria [8]. A limitation of the WHO diagnostic set is that no criteria were specified to exclude epithelial dysplasia from the diagnosis of OLP. For this reason, according to Ismail SB et al., a significant percentage of patients who developed carcinoma from oral lichen planus were actually misdiagnosed cases of other lichenoid lesions with higher malignant potential [9]. In addition, a distinction between idiopathic OLP and other OLLs, such as contact or drug-induced lichenoid reactions, cannot be made based on these criteria. Furthermore, studies have found that application of the WHO criteria was associated with the highest rate of clinicopathological discrepancy [6] and inter- and intraobserver variability in the histological assessment of oral lichen planus [10].

In 2003, Van der Meij and van der Waal proposed some modifications to the WHO criteria for OLP [6]. The authors retained some of the WHO criteria, namely liquefaction degeneration of the basal cells and a dense band-like inflammatory infiltrate composed mainly of lymphocytes and confined to the superficial lamina propria. The novelty of this modified diagnostic set is the inclusion of an additional criterion to confirm the absence of epithelial dysplasia. Along with this, the authors introduced the term “histopathologically compatible with OLP” for all cases that do not meet all of the listed criteria. This definition points to an oral lichenoid lesion other than OLP. Later, Rad M. et al. found increased clinicopathological agreement in the diagnosis of OLP when applying van der Meij’s modified criteria compared to those of the WHO [11]. However, some of the typical features of OLP that stem from important mechanisms of disease pathogenesis, such as lymphocyte migration into the epithelium, were not addressed in the modified 2003 WHO criteria.

The most recent update to the diagnostic criteria for OLP was made by the American Academy of Oral and Maxillofacial Pathology (AAOMP) in 2016 and includes a set of clinical and histological criteria [5]. Lymphocyte exocytosis and lack of verrucous epithelium architectural change are the new criteria added to those cited by Van der Meij and van der Waal. The authors stated that the diagnosis of OLP requires fulfillment of all proposed clinical and histopathological criteria.

The aim of the study was to assess the reliability of the AAOMP histological criteria in diagnosing OLP.

## 2. Materials and Methods

Research Focus Questions

- What is the reproducibility of the histological diagnoses of OLP when applying the different sets of diagnostic criteria?
- Do histological sections, once diagnosed as OLP, meet all diagnostic criteria proposed by the American Academy of Oral and Maxillofacial Pathology?

And if not,

- Which features of OLP are found consistently in all histological sections?

Study Design

This was a retrospective study that analyzed data obtained from histological reports and histological sections from patients with OLP who were diagnosed and biopsied at the Department of Periodontology and Oral Mucosal Diseases, Faculty of Dental Medicine, MU-Plovdiv between 1 January 2013 and 30 June 2016. The initial histological examination that confirmed the diagnosis of OLP was performed at the Department of General and Clinical Pathology of MU-Plovdiv. The study was conducted in accordance with the Declaration of Helsinki and was approved by the Ethics Committee of the Medical University of Plovdiv (R3716/7 October 2014). Histopathological reports were used to extract information regarding the following variables: biopsy site, clinical form of OLP and demographic characteristics of the patients included in the study. Existing hematoxylin and eosin (H&E)-stained histological slides, stored in the pathology laboratory, were revised by a single experienced pathologist who was blinded to the evaluation done retrospectively for this study in order to determine the interobserver variability in the histological diagnosis of OLP. Because the criteria used by the first evaluator were not stated in the histopathology reports, no requirements were placed on the pathologist assigned to the present study regarding the set of diagnostic criteria during this second revision of the diagnosis. After completion of the histological examination, the pathologist was asked to specify the criteria used. The same evaluator was then asked to utilize the newly proposed diagnostic set of the AAOMP while assessing slides, categorizing each criterion listed there as present (P) or absent (A).

Research Materials

- Archived histopathological reports of patients with OLP, biopsied at the Department of Periodontology and Oral Mucosal Diseases, Faculty of Dental Medicine, MU-Plovdiv, between 2013 and 2016. Only patients for whom the clinical diagnosis of OLP was confirmed by the histological examination were included in the study.
- Corresponding hematoxylin and eosin (H&E)-stained histological slides of the included patients.

Histological Analysis

All histological slides selected for revision were analyzed by light microscopy (Leica DM 2000 LED, Leica Microsystems, Wetzlar, Germany). The diagnoses made by the pathologist during this second revision were categorized as “evident OLP”, “compatible with OLP” or “other mucosal lesion” and compared with the original. The criteria used by the pathologist correspond to the WHO criteria. The same evaluator was then asked to score all histological slides for the presence (P) or absence (A) of the following morphological parameters: predominantly lymphocytic infiltrate confined to the epithelium–lamina propria interface; basal cell liquefactive (hydropic) degeneration; lymphocytic exocytosis; epithelial dysplasia; verrucous epithelial architectural change. Based on the results obtained, the number of cases that fulfill the criteria proposed by the American Academy of Oral and Maxillofacial Pathology was calculated.

## 3. Results

Over a period of 4 years (2013–2016), a total of 43 patients with a clinically suspected diagnosis of OLP were biopsied at the Department of Periodontology and Oral Mucosal Diseases. In 34 of them, the diagnosis was histologically confirmed. According to the data filled in the histopathological reports, the male to female ratio was 6:28, and age ranged 23–78 years old. The age distribution was as follows: <30 years (2); 31–40 (2); 41–50 (11); 51–60 (7); and >61 (12). The buccal mucosa was the most common site for biopsy (25), followed by gingiva (7) and tongue (2). Diagnoses included 14 patients with reticular form, 1 with papular, 2 with plaque-like, 7 with atrophic, 9 with erosive and 1 with bullous form of OLP.

Of the 34 histological slides initially (at the first histological evaluation) diagnosed with OLP, 31 were categorized as “evident OLP” and another 3 as “compatible with OLP” by the second pathologist. No slides were diagnosed as “other mucosal lesion”. The criteria used by the pathologist correspond to the WHO criteria and were as follows: presence of hyperkeratosis and a band-like inflammatory infiltrate consisting predominantly of lymphocytes, presence or absence of acanthosis, epithelial atrophy, Civatte bodies or basal cell liquefaction degeneration. Agreement between the first and second evaluators regarding the diagnosis of OLP was found in 91.1% of the samples.

The morphological findings observed in the studied samples are shown in Table 1. From the diagnostic criteria proposed by the American Academy of Oral and Maxillofacial Pathology, basal cell liquefactive (hydropic) degeneration was found in 35.2% (*n* = 12), lymphocytic exocytosis in 32.3% (*n* = 11) and mild epithelial dysplasia in 2.9% (*n* = 1) of the samples. A band-like predominantly lymphocytic infiltrate, confined to the epithelium–lamina propria interface, was present in 55.8% (*n* = 19) of the sections, and in another 26.4% (*n* = 9), the infiltrate was polymorphic, composed of lymphocytes and plasma cells (with or without some neutrophils and eosinophils). In 29.4% (*n* = 10) of the samples, deep/perivascular infiltrate was demonstrated. There were no cases of verrucous epithelial architectural change. None of the histological features of OLP were consistently found in all histological sections.

**Figure 1:**
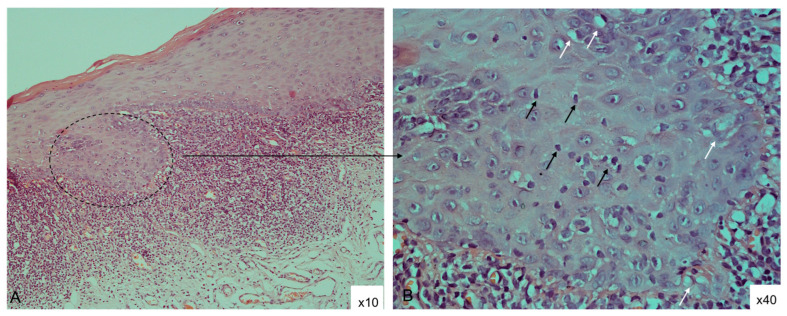
Tissue section diagnosed as OLP based on the AAOMP histological criteria, H&E staining: (**A**): ×10 magnification, epithelial hyperkeratosis and acanthosis and dense band-like inflammatory infiltrate of lymphocytes in the superficial lamina propria; (**B**): same specimen ×40 magnification, hydropic degeneration (white arrows), lymphocytic exocytosis (black arrows) and absence of epithelial dysplasia.

Analysis of the aforementioned data showed that only 17.6% (*n* = 6) of the samples fulfill all histological diagnostic criteria proposed by the American Academy of Oral and Maxillofacial Pathology (simultaneous presence of predominantly lymphocytic infiltrate confined to the epithelium-lamina propria interface; basal cell liquefactive (hydropic) degeneration; lymphocytic exocytosis; absence of epithelial dysplasia; absence of verrucous epithelial architectural change) (Figure 1).

**Figure 2:**
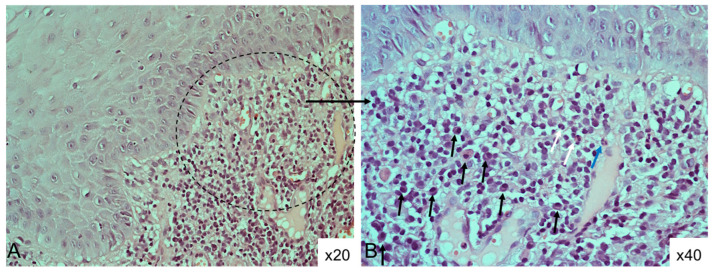
Histological section (H&E staining) diagnosed as OLP based on the 1978 WHO criteria, which does not meet the AAOMP diagnostic criteria, due to (**A**): absence of hydropic degeneration and lymphocytic exocytosis in the epithelium (×20 magnification) and (**B**): presence of polymorphic inflammatory infiltrate in the lamina propria; plasma cells (black arrows), eosinophils (white arrows), neutrophil (blue arrow); (×40 magnification).

Reproducibility of the histological diagnosis of OLP was achieved in only 19.3% of the cases when applying the 1978 WHO criteria versus the newly proposed AAOMP criteria. Figure 2 illustrates a case of discrepancy in the diagnoses of oral lichen planus as a result of the different diagnostic sets used.

## 4. Discussion

Oral lichen planus is considered a clinico-pathological diagnosis [6]. Therefore, biopsy should be taken from all patients with clinical manifestations suggestive of OLP, not only to confirm the diagnosis but also to exclude malignancy. However, especially in cases with the classic reticular form of the disease, histological examination is often omitted [3]. This may explain the relatively low number of histological sections from OLP patients over a 4-year period analyzed in the present study. A review of the data from the archive of the Department of Periodontology and Oral Mucosal Diseases showed that out of a total of 81 patients clinically diagnosed with OLP during this period, only 43 had a biopsy performed.

Agreement on the histological diagnosis of OLP between the pathologist who had performed the initial examination and the second pathologist who was asked to review the same tissue sections for the purposes of the present study was achieved in 91.1% of cases. WHO criteria were used by the second observer. The criteria applied during the first examination were not reported in the histopathology reports we reviewed. However, since we intentionally limited the observation period to before 30 June 2016, it is unlikely that the pathologist used AAOMP criteria, as these were first introduced in September 2016. Therefore, we believe that the initial and second revised diagnoses of OLP were made based on the same criteria.

In 1978, the WHO Collaborating Centre for Oral Precancerous Lesions published a number of histological features commonly observed in tissue sections from patients with OLP to be used as diagnostic criteria [8]. However, no requirement was made to fulfill all these criteria. Thus, OLP specimens demonstrating all the typical features of the disease are relatively easy to diagnose, but in cases covering part of the criteria, the diagnosis is made based on the subjective judgment of the pathologist. The difficulty comes from the fact that most of the listed histological features are not specific to OLP but can also be found in other diseases. For example, interface mucositis (band-like chiefly lymphocytic infiltrate in superficial lamina propria) is also present in lupus erythematosus, drug-induced and contact lichenoid reactions, in some cases of proliferative verrucous leukoplakia, oral epithelial dysplasia and squamous cell carcinoma. Liquefaction degeneration of basal keratinocytes could be seen in graft-versus-host disease (GVHD), contact and drug-induced lichenoid reactions and lupus erythematosus. Civatte bodies are a characteristic finding in chronic ulcerative stomatitis (CUS), lupus erythematosus (LE), GVHD, drug-induced lichenoid reactions, etc. [5,12,13]. Another disadvantage of the WHO diagnostic set is that the presence of epithelial dysplasia is not listed as an exclusion criterion. In 1985, Krutchkoff and Eisenberg introduced the term “lichenoid dysplasia” for lesions that histologically demonstrate epithelial dysplasia in combination with band-like chronic inflammatory infiltrate in the superficial lamina propria and/or other lichenoid features (saw-tooth rete pegs, Civatte bodies and basal cell degeneration) [14,15]. In a study by Fitzpatrick et al., band-like inflammatory infiltrate and basal cell degeneration (lichenoid features) were found in 74% and 30% of oral epithelial dysplasia (OED) specimens, respectively [16]. Thus, dysplastic lesions are often diagnosed as OLP. According to current understanding, the use of the term “lichenoid dysplasia” is the main reason for the misdiagnosis of OED (being a separate entity) as OLP. Therefore, the workgroup from the international workshop on nomenclature and classification convened by the WHO Collaborating Centre for Oral Cancer (2020) [7] recommended that the term “lichenoid dysplasia” be removed and stated that if dysplasia is present, the diagnosis should be oral epithelial dysplasia with lichenoid features. In the present study, epithelial dysplasia was found in one specimen (Table 1). However, both the first and second pathologists diagnosed the case as oral lichen planus, which again confirms the assumption that both used the WHO criteria.

The newly proposed diagnostic set of the American Academy of Oral and Maxillofacial Pathology is based on the knowledge accumulated over the years regarding the distinguishing characteristics of some of the most common “OLP mimics” [5]. According to the authors, the use of the listed criteria will make the OLP patient group a more homogeneous population of idiopathic OLP for future research, which would increase the validity of any statements regarding the pathogenesis and malignant potential of the disease.

Liquefaction, also known as hydropic degeneration of the basal cells, is a pathological edema of the keratinocytes due to an increased membrane permeability of the damaged cell. The influx of water in hydropic degeneration dilutes the cytoplasm, separates cell organelles and stretches the cell [17]. Microscopically, the affected keratinocytes appear greatly enlarged with pale and homogenized cytoplasm, creating in places the impression of optically empty cells (Figure 1). In the present study, hydropic degeneration was found in only 35.2% (*n* = 12) of the OLP tissue sections. Similar results were reported by Sanches et al., who observed basal layer degeneration in 39.6% (*n* = 19) of the specimens (*n* = 48) from OLP lesions analyzed in their study [3].

Migration of lymphocytes into the overlying epithelium, known as lymphocytic exocytosis, is a newly added criterion for the diagnosis of OLP by the AAOMP [5]. The general consensus regarding the pathogenesis of the disease is that it is an immune-mediated condition in which CD8+ T-lymphocytes destroy basal keratinocytes by activating the cell death program (apoptosis). Jungell et al. conducted an immunoelectron microscopic study to demonstrate that most of the CD8+ cells were located intraepithelially, adjacent to apoptotic keratinocytes [18]. Therefore, the AAMOP working group considered it justified to observe lymphocyte exocytosis in all cases of OLP and to include it as a mandatory diagnostic criterion. In addition, according to the authors, this finding may help distinguish OLP from other oral lichenoid conditions, such as MMP, because the immune aggression there is directed against adhesion molecules in the basement membrane zone and lymphocytes are not usually seen in the epithelium. However, lymphocyte exocytosis was not a consistent finding among the specimens we analyzed. In particular, it was detected in only 32.3% (*n* = 11) of them (Figure 1).

The detection of Civatte bodies in the basal layer is not listed as a criterion in the newly proposed AAOMP diagnostic set. Civatte (also known as colloid or hyaline) bodies represent anucleated remnants of epithelial cells formed as a result of an activated process of programmed cell death (apoptosis). In recent years, the thesis of pathologically enhanced apoptosis being present in OLP has been disputed [19]. One of the reasons for this is the reported low number of Civatte bodies [19]. Of note, in our study, colloid bodies were found in only three cases (8.8%).

The absence of verrucous epithelial architectural change was added by the AAOMP as a diagnostic criterion in an attempt to differentiate OLP and proliferative verrucous leukoplakia (PVL), as these two entities share similar clinical and histopathological features [5]. Verrucous architecture is identified by a papillary configuration of the spinous cell layer accompanied by variable levels of mucosal surface corrugation [5]. Such changes were not observed in any of the samples we analyzed.

According to the AAOMP working group, the presence of epithelial dysplasia in the specimen excludes the diagnosis of oral lichen planus [5]. Therefore, “OLP with dysplasia” cannot be the pathologist’s conclusion at the first histological examination of a patient. This diagnosis can be made during a follow-up histological examination if signs of malignancy have appeared in the evolution of the initially non-malignant OLP lesion. In the present study, epithelial dysplasia was observed in one specimen. Applying the AAOMP diagnostic criteria, the latter cannot be diagnosed as oral lichen planus.

Last but not least, the mononuclear, predominantly lymphocytic, band-like infiltrate in the superficial lamina propria (interface mucositis), being one of the most characteristic microscopic findings of OLP, is also listed as a mandatory diagnostic criterion by the AAOMP. However, Sanches et al. reported an absent/mild inflammatory infiltrate in 25% of the OLP samples (*n* = 48) they analyzed [3]. In the present study, inflammatory infiltrate in the epithelium–lamina propria interface was found in all cases. However, in 26.4% (*n* = 9) of them, the infiltrate was polymorphic (composed of lymphocytes, plasma cells, eosinophils and/or neutrophils) (Figure 2), and in 29.4% (*n* = 10), a deep/perivascular infiltrate was additionally observed. A dense lymphocytic infiltrate forming tertiary follicles was found in one specimen. All of these findings—diffuse lymphocytic infiltrate mixed with plasma cells and eosinophils extending deeper into the lamina propria, focal perivascular infiltrate and tertiary lymphoid follicles—have been repeatedly highlighted in the literature as distinguishing features of oral lichenoid reactions (OLRs) [12,20]. The latter term refers to lesions that are clinically and histologically similar to those of OLP but have an identifiable causative factor (contact OLR, drug-induced OLR and GVHD-associated OLR), the removal of which results in regression of the lesions. Differentiating OLR from idiopathic OLP is a diagnostic challenge. Unfortunately, even this set of strict criteria may not be helpful in distinguishing these two entities, as OLRs often show microscopic features that fulfill all of the histopathological criteria proposed by the AAOMP. However, the authors recommend pathologists refrain from diagnosing OLP if eosinophils or a perivascular lymphoplasmacytic infiltrate in the deep lamina propria are present, although these findings are not formally listed as exclusion criteria in their diagnostic set (Figure 2) [5]. In the present study, one of the six specimens meeting all AAOMP diagnostic criteria demonstrated a mixed (lymphocyte and plasma cell) infiltrate, and another one demonstrated additional deep/perivascular inflammation. It is worth noting that the clinical type (reticular vs. erosive) and anatomical site (buccal mucosa vs. gingiva) should also be considered when making a histological diagnosis, as both may influence the subsets of inflammatory cells in the infiltrate [5]. For example, areas of erosion and ulceration typically demonstrate superimposed inflammation, resulting in a mixed inflammatory infiltrate rich in neutrophils, and in gingival OLP lesions, the infiltrate is often mixed with plasma cells due to concomitant gingivitis or periodontitis [5,12]. Of note, none of the tissue sections demonstrating a mixed with plasma cell and/or neutrophils infiltrate in the present study were taken from gingiva, and erosion was not found in any of them.

In summary, the results obtained in the present study raised the following concerns: 1. Nine years after the introduction of the AAOMP diagnostic criteria for OLP, their validity has not been confirmed by serious randomized trials, and they have not been adopted by the WHO, which is why a proportion of pathologists (as demonstrated in the present study) still use the WHO criteria from 1978. 2. Strict adherence to the proposed diagnostic set excludes a large number of patients who would otherwise be diagnosed as OLP cases. In the present study, only 6 of 31 specimens, for which two independent pathologists agreed on the diagnosis of OLP, fulfilled all AAOMP histological criteria. These results question the reliability of the proposed diagnostic set. 3. Further studies with an increased number of OLP cases are needed to assess the frequency of observation of the different histological variables to confirm their significance for the diagnosis of the disease. If the results we presented are confirmed by studies with an increased sample size, it would mean that none of the AAOMP histological criteria can be defined as mandatory for the diagnosis of OLP. In this case, a proposal for a new diagnostic set that distinguishes essential (mandatory) versus desirable criteria would be necessary. 4. To date, regardless of the set of diagnostic criteria used, the diagnosis of OLP cannot be made solely on the basis of histological examination. Cases with a histological diagnosis “compatible with OLP” and those with clinic–pathological discordance require monitoring of the evolution of the condition and therapeutic response, control biopsy examination and the implementation of additional diagnostic methods such as direct immunofluorescence, allergic (Patch) test, etc.

The small sample size and single observer assessment of the AAOMP criteria should be noted as limitations of the present study.

## 5. Conclusions

In the present study, a discrepancy was found in the histological diagnoses of oral lichen planus based on the WHO criteria versus the newly proposed AAOMP diagnostic set. The reason for this was that strict adherence to the AAOMP guidelines excluded a large number of OLP cases. The histological features listed by the Academy as mandatory diagnostic criteria were observed in a relatively low percentage of the OLP tissue sections we analyzed, and none of them were consistently found in all histological sections. Further studies are needed to assess the validity of the AAOMP diagnostic criteria.

## Figures and Tables

**Table 1:** Frequency of observation of the different histological variables of OLP.

Variable	Total Sample (*n* = 34)	
	Frequency (%)	(*n*)
Hyperkeratosis	91.1%	31
Acanthosis	85.2%	29
Epithelial atrophy	17.6%	6
Erosion	2.9%	1
Epidermo-dermal detachment	23.5%	8
Basal layer hydropic degeneration	35.2%	12
Lymphocytic exocytosis	32.3%	11
Epithelial dysplasia	2.9%	1
Verrucous epithelialarchitectural change	0%	0
Civatte bodies	8.8%	3
Saw-tooth rete ridges	35.2%	12
Inflammatory infiltrate	100%	34
band-like, lymphocytic infiltrate confined only to the epithelium–lamina propria interface	55.8%	19
polymorphic inflammation (Ly, plasma cells, Eo, Neu)	26.4%	9
deep/perivascular	29.4%	10
dense lymphocytic infiltrate forming tertiary follicles	2.9%	1

## Data Availability

The data that support the findings of this study are available from the corresponding author, [M.Z.M.], upon reasonable request.

## References

[1] Manchanda Y., Rathi S.K., Joshi A., Das S. (2023). Oral Lichen Planus: An Updated review of etiopathogenesis, clinical presentation, and management. Indian. Dermatol. Online J..

[2] Vičić M., Hlača N., Kaštelan M., Brajac I., Sotošek V., Massari L.P. (2023). Comprehensive Insight into Lichen Planus Immunopathogenesis. Int. J. Mol. Sci..

[3] Sanches A.C.B., Pires A.L.P.V., Medrado A.R.A.P., De Almeida Reis S.R., Freitas V.S., Martins G.B. (2022). Oral Lichen planus: Associations between histomorphometric characteristics and white and red lesions. Head Neck Pathol..

[4] Mutafchieva M.Z., Draganova-Filipova M.N., Zagorchev P.I., Tomov G.T. (2018). Oral Lichen Planus—Known and Unknown: A Review. Folia Med..

[5] Cheng Y.-S.L., Gould A., Kurago Z., Fantasia J., Muller S. (2016). Diagnosis of oral lichen planus: A position paper of the American Academy of Oral and Maxillofacial Pathology. Oral Surg. Oral Med. Oral Pathol. Oral Radiol..

[6] Van Der Meij E.H., Van Der Waal I. (2003). Lack of clinicopathologic correlation in the diagnosis of oral lichen planus based on the presently available diagnostic criteria and suggestions for modifications. J. Oral Pathol. Med..

[7] Warnakulasuriya S., Kujan O., Aguirre-Urizar J.M., Bagan J.V., González-Moles M.Á., Kerr A.R., Lodi G., Mello F.W., Monteiro L., Ogden G.R. (2020). Oral potentially malignant disorders: A consensus report from an international seminar on nomenclature and classification, convened by the WHO Collaborating Centre for Oral Cancer. Oral Dis..

[8] Kramer I.R., Lucas R.B., Pindborg J.J., Sobin L.H. (1978). Definition of leukoplakia and related lesions: An aid to studies on oral precancer. Oral Surg. Oral Med. Oral Pathol..

[9] Ismail S.B., Kumar S.K.S., Zain R.B. (2007). Oral lichen planus and lichenoid reactions: Etiopathogenesis, diagnosis, management and malignant transformation. J. Oral Sci..

[10] Van Der Meij E.H., Reibel J., Slootweg P.J., Van Der Wal J.E., De Jong W.F.B., Van Der Waal I. (1999). Interobserver and intraobserver variability in the histologic assessment of oral lichen planus. J. Oral Pathol. Med..

[11] Rad M., Hashemipoor M.A., Mojtahedi A., Zarei M.R., Chamani G., Kakoei S., Izadi N. (2009). Correlation between clinical and histopathologic diagnoses of oral lichen planus based on modified WHO diagnostic criteria. Oral Surg. Oral Med. Oral Pathol. Oral Radiol. Endod..

[12] Müller S. (2017). Oral lichenoid lesions: Distinguishing the benign from the deadly. Mod. Pathol..

[13] Müller S. (2011). Oral Manifestations of Dermatologic Disease: A focus on lichenoid lesions. Head Neck Pathol..

[14] Krutchkoff D.J., Eisenberg E. (1985). Lichenoid dysplasia: A distinct histopathologic entity. Oral Surg. Oral Med. Oral Pathol..

[15] Speight P.M., Khurram S.A., Kujan O. (2017). Oral potentially malignant disorders: Risk of progression to malignancy. Oral Surg. Oral Med. Oral Pathol. Oral Radiol..

[16] Fitzpatrick S.G., Honda K.S., Sattar A., Hirsch S.A. (2014). Histologic lichenoid features in oral dysplasia and squamous cell carcinoma. Oral Surg. Oral Med. Oral Pathol. Oral Radiol..

[17] Miller M.A., Zachary J.F. (2017). Mechanisms and morphology of cellular injury, adaptation, and death. Elsevier eBooks.

[18] Jungell P., Konttinen Y.T., Nortamo P., Malmström M. (1989). Immunoelectron microscopic study of distribution of T cell subsets in oral lichen planus. Scand. J. Dent. Res..

[19] Bascones C., Gonzalez-Moles M.A., Esparza G., Bravo M., Acevedo A., Gil-Montoya J.A., Bascones A. (2005). Apoptosis and cell cycle arrest in oral lichen planus. Arch. Oral Biol..

[20] Kalele K., Sarode S.C., Sarode G.S. (2012). Oral Lichenoid Reaction: A Review. Internat J. Oral Maxillofac. Pathol..

